# Development of Pedigree Classification Using Microsatellite and Mitochondrial Markers for Giant Grouper Broodstock (*Epinephelus lanceolatus*) Management in Taiwan

**DOI:** 10.3390/md12052397

**Published:** 2014-04-30

**Authors:** Hsiao-Che Kuo, Hao-Hsuan Hsu, Chee Shin Chua, Ting-Yu Wang, Young-Mao Chen, Tzong-Yueh Chen

**Affiliations:** 1 Laboratory of Molecular Genetics, Institute of Biotechnology, College of Bioscience and Biotechnology, National Cheng Kung University, Tainan 70101, Taiwan; E-Mails: shoujer@gmail.com (H.-C.K.); samuel0801@msn.com (H.-H.H.); cheeshinc@gmail.com (C.S.C.); nnigdd@gmail.com (T.-Y.W.); ymc868@yahoo.com.tw (Y.-M.C.); 2 Translational Center for Marine Biotechnology, National Cheng Kung University, Tainan 70101, Taiwan; 3 Agriculture Biotechnology Research Center, National Cheng Kung University, Tainan 70101, Taiwan; 4 University Center of Bioscience and Biotechnology, National Cheng Kung University, Tainan 70101, Taiwan; 5 Research Center of Ocean Environment and Technology, National Cheng Kung University, Tainan 70101, Taiwan

**Keywords:** giant grouper, inbreeding, mitochondria, microsatellite

## Abstract

Most giant groupers in the market are derived from inbred stock. Inbreeding can cause trait depression, compromising the animals’ fitness and disease resistance, obligating farmers to apply increased amounts of drugs. In order to solve this problem, a pedigree classification method is needed. Here, microsatellite and mitochondrial DNA were used as genetic markers to analyze the genetic relationships among giant grouper broodstocks. The 776-bp fragment of high polymorphic mitochondrial D-loop sequence was selected for measuring sibling relatedness. In a sample of 118 giant groupers, 42 haplotypes were categorized, with nucleotide diversity (π) of 0.00773 and haplotype diversity (HD) of 0.983. Furthermore, microsatellites were used for investigation of parentage. Six out of 33 microsatellite loci were selected as markers based on having a high number of alleles and compliance with Hardy-Weinberg equilibrium. Microsatellite profiles based on these loci provide high variability with low combined non-exclusion probability, permitting practical use in aquaculture. The method described here could be used to improve grouper broodstock management and lower the chances of inbreeding. This approach is expected to lead to production of higher quality groupers with higher disease resistance, thereby reducing the need for drug application.

## 1. Introduction

The numbers of domesticated and farmed fish are increasing to facilitate feeding of the burgeoning human population [1]. More than 50 different species of grouper inhabit the tropical waters around Taiwan and some have been farmed since 1979. Among them, giant grouper is the most valuable grouper species in Taiwan. Although key aquaculture techniques on an industrial scale have been well established, outbreaks of various diseases remain a major unsolved problem [2,3,4]. However, most grouper broodstocks are second generation from wild-caught parent fish [5]. The larvae (third generation) cultivated in the fish farm for sale are derived from those broodstocks, and some of these larvae may be kept and used as broodstocks. The mixture of second- and third-generation broodstocks in the same pond means that the larvae (fourth generation) will be derived by inbreeding. Inbreeding is expected to lead to the appearance of defective recessive alleles that will reduce the trait quality and survival rate, resulting in growth depression and sensitive to environmental stress [6,7,8,9]. To solve this problem, a systematic broodstock management platform that can track the family tree by using genetic markers must be established [10]. An ideal genetic marker would be one that incorporates aspects of variability, heritability, stability, and accessibility during identification [11]. Microsatellite DNA is commonly used as a genetic identification tool due to its high polymorphism, co-dominant features and neutral mutation [12,13]. Microsatellite markers can reveal the genetic inheritance of an individual within a population [14,15], given that there is a low probability of different and non-related individuals exhibiting the same microsatellite pattern [16]. Besides, the use of the mitochondrial D-loop has been proposed as a genetic marker as well; this segment of the mitochondrion, a maternally inherited genetic material, exhibits high variability and can be used to identify sibling relationship within a population [17].

Microsatellite and mitochondria markers have been used previously for tracking the genetic history of groupers [18,19,20,21,22]. Although there are many successful applications of both microsatellites and mitochondrial D-loop as genetic markers for identification of individual groupers, the management of giant grouper broodstocks in Taiwan still lacks a practical methodology. In this study, we used both microsatellite markers and mitochondrial D-loop sequences to develop a system for identification of parentage and sibling relationships. Establishment of these genetic markers is expected to ensure trait quality of broodstocks while not only preventing inbreeding depression like physical and health defects but also reducing drug and therapeutant use in aquaculture.

## 2. Results and Discussion

### 2.1. Analysis of Mitochondrial D-Loop Region

Within the 776-nt D-loop fragment examined here, 56 nucleotides exhibited variations, including insertion and deletion, among the 118 giant grouper broodstocks (Table 1). Among those 56 nucleotide variations, 42 haplotypes can be identified (a single mutation site correspond to a single genotype). The nucleotide diversity (π) was 0.00773, and the haplotype diversity (HD) was 0.983 (Table 1). The genetic distance of haplotypes ranged between 0.215 and 0.0013, with an average of 0.0079 (Table 1). From 118 samples, the variation of nucleotides ranged between 16.684 and 1.0088 (average = 6.1304). These values indicate that the D-loop region used in this study is reliable for biogeographic analysis and sibling relationship determination.

**Table 1:** Mitochondrial D-loop-based genetic diversity of giant groupers in Taiwan aquaculture industry.

Parameter to the Genetic Diversity	
Farms	3
Samples	118
Haplotypes	42
Length (base pair)	776
Nucleotide variations	56
Haplotype diversity(Hd)	0.983
Nucleotide diversity(π)	0.00773
Maximum distance between each two haplotypes	0.0215
Minimum distance between each two haplotypes	0.0013
Mean distance between each two haplotypes	0.0079

In other developed systems, either of two distinct genetic markers (the mitochondrial D-loop and multiallelic microsatellites) has been used to assess variation within populations and to identify individuals. Low variation in D-loop nucleotide sequences of this fish species from our investigation suggested that the broodstock inbreeding level in Taiwan is high.

### 2.2. Analysis of Giant Grouper Microsatellite Loci

Examination of the results for microsatellite loci in giant grouper (Table 2) revealed that there were only 21 amplifiable sequences. Those loci were analyzed for allele number, allele distribution, expected heterozygosity (EH), and observed heterozygosity (OH) (Table 2). Among those loci, six were shortlisted based on their high heterozygosity and variability, properties that were expected to permit reliable parentage relationship determinations with high distinguishability and low error probability. These six shortlisted loci included Efu19 (EH, 0.746063; OH, 0.8125), ELMS009 (EH, 0.77842; OH, 0.875), ELMS015 (EH, 0.83157; OH, 0.640625), RH_CA_2 (EH, 0.822466; OJ, 0.890625) and RH_GATA_3 (EH, 0.845349; OH, 0.828125) (Table 2) are all carry a high number of alleles and comply with Hardy-Weinberg Equilibrium.

**Table 2:** Primers used for characterization of microsatellite loci in giant grouper.

Locus *	Accession Number	Repeat Motif	Primer Sequences (5′→3′)	Allele Size	Allele Number	Expected Heterozygosity (EH)	Observed Heterozygosity (OH)
Efu02 ^a^	EU016533	(CA)*n*	F: CTGTCTCAGCTGATTTATGG	345–371	10	0.667815	0.484375
			R: TTTACAGTCTCGTGGTTTCA				
Efu06 ^a^	EU016535	(GACA)*n*	F: CATTGTCATTGTTGCTGTTTCTGTC	308	1	0	0
			R: CCCTTTGGCCAATTGATGTGAT				
Efu08 ^a^	EU016537	(CA)*n*	F: TGGAGAAGCCTGTAGATTATTGTG	292–330	9	0.783465	0.796875
			R: AAGCAGGAGAGGAGTTGAAGGAGT				
Efu18 ^a^	EU016543	(CA)*n*	F: ACTGGCTCCCTTCTGTTCC	370–384	2	0.361713	0.4375
			R: ATTGCCACCATCGCTACC				
Efu19 ^a^	EU016544	(CA)*n*	F: GGGCGGTAACCTCTCCAG	93–115	7	0.746063	0.8125
			R: AGCAGCAACACCTTCTTCTCA				
Efu41 ^a^	EU016545	(CA)*n*	F: CAGCACGCAGTTTAATTTACCAG	243–249	2	0.353223	0.390625
			R: CAGGACCCGAGCTTCAGAA				
ELMS009 ^b^	EF607131	(CA)*n*	F: TTCCACAGCAATTAGCAGCA	260–278	8	0.77842	0.875
			R: TTTCCTCCCACAGTCCAAAG				
ELMS015 ^b^	EF607136	(TG)*n*	F: AAGCTGAGCCGAATTTTTCA	335–369	12	0.83157	0.640625
			R: GCTCCTCGTGTTTCCGATTA				
Epaw3 ^c^	EU684479	(GT)*n*	F: GTCGTGTCTGTGACCATGAG	72–76	2	0.503445	0
			R: TAAGGAGGGGGCTAAATGAT				
Epaw6 ^c^	EU684482	(GT)*n*	F: ATGGTGTGGGAAAAGAGAGT	146–233	5	0.619218	0.453125
			R: TTGTTTCAGGACAAGTGAGC				
Epaw19 ^c^	EU684495	(GT)*n*	F: AGGTGGCTTGTGTGTGTATT	243–247	3	0.215428	0.234375
			R: GCTTCCTTGACTGCTATGAC				
Epaw34 ^c^	EU684510	(TG)*n*	F: ACAGCACCTCTACCATGAAC	224–248	3	0.212968	0.078125
			R: CGTCCCCGTATATATCTCTG				
CA-2 ^d^	AF539606	(CA)*n*	F: GACTTGATTCAGCAAAATAAAGATG	150–262	6	0.350271	0.0625
			R: AGAGACGGTGCCAGTAAATGAA				
CA-3 ^d^	AF539605	(CA)*n*	F: ATGTGACACGTTGACAGGCAAGT	300	1	0	0
			R: GACCTTGATATTTTCATTGCTTG				
CA-6 ^d^	AF539608	(CA)*n*	F: GTGTTGCTGGGGTTACTAATGAAG	266–290	4	0.495325	0.5
			R: TTAGACACATTGTCACGATGGTCC				
RH_CA_1 ^e^	DQ223785	(CA)*n*	F: CGAGATAAGCCCTGGTGAAA	376–388	3	0.452879	0.46875
			R: AGTCCCGATGTGGTAACGAG				
RH_GATA_2 ^e^	DQ223791	(GATA)*n*	F: CTCGACAGTGGACAAGGTCA	132	1	0	0
			R: AAGGGCATGATGGGAAATG				
RH_CA_2 ^e^	DQ223785	(CA)*n*	F: CTCGTTACCACATCGGGACT	135–175	13	0.822466	0.890625
			R: AACACTGGCTGGTTTGCACT				
RH_GATA_3 ^e^	DQ223790	(GATA)*n*	F: GGGCAATTTGGTTCTTCACA	225–273	10	0.845349	0.828125
			R: TGTCAATGCCACAGGATACA				
RH_CA_7 ^e^	DQ223786	(CA)*n*	F: CAGAAACATCTCCCCCAAAA	259–335	12	0.639887	0.34375
			R: CTGGCAGAGCAATTAGAGGC				
RH_CA_8 ^e^	DQ223787	(CA)*n*	F: AGTTGCCCAGGTTACACGAG	219–227	4	0.554995	0.515625
			R: TTGGGTCCTGGCATTTAGAG				

* The loci listed here represent 21 microsatellite loci selected from previous studies of various species: (i) Tiger grouper: Efu02, Efu06, Efu08, Efu18, Efu19, Efu41; (ii) Giant grouper: ELMS009, ELMS015; (iii) Banded grouper: Epaw3, Epaw6, Epaw19, Epaw34; (iv) Hawaiian grouper: CA-2, CA-3, CA-6; (v) Red-spotted grouper: RH_CA_1, RH_CA_2, RH_CA_7, RH_CA_8, RH_GATA_2, RH_GATA_3; The references used for microsatellite loci primer design are ^a^ Lo and Yue, 2008 [23]; ^b^ Zeng *et al.*, 2008 [24]; ^c^ Zhao *et al.*, 2009 [25]; ^d^ Rivera *et al.*, 2003 [21]; ^e^ Ramírez *et al.*, 2006 [26], respectively.

By applying these six loci to a subset of 64 fish, the first parent non-exclusion probability is 0.01138738 (or 98.86% accurate) which we can identify the parentage relationship without knowing the genotypes of both parent fish. The non-exclusion probability (second parent) will lower (0.00089627 or 99.91% accurate) when one of the parent fish genotype is known (Table 3). It is important to obtain low combined non-exclusion probability (first and second parent) which means the combination of all six loci is predicted to determine parentage with low chance of misjudgment. 

Knowing the origin of the broodstock can help to preclude collection of genetically closely related fish. We collected the samples from farms known to constantly replenish the broodstock from wild-caught fish or by purchasing small numbers of giant groupers from each of multiple different fish farms. This approach is recommended, since it can increase genetic variation within the fish population.

**Table 3:** Paternity exclusion probabilities based on six selected microsatellite loci.

Paternity Exclusion Probabilities	
Combined non-exclusion probability (first parent)	0.01138738
Combined non-exclusion probability (second parent)	0.00089627

The observed large numbers of haplotypes (from the mitochondrial D-loop sequence) and high allele numbers (among the six multiallelic microsatellite loci) indicate that our samples were derived from different genetic pools. Nevertheless, the present study analyzed only a small number of giant groupers; the sample sizes of future studies will affect the determination of the number of D-loop haplotypes, number of microsatellite loci alleles, and their combined non-exclusive probabilities. Analysis of mitochondrial D-loop alone may have limitations. Offspring from a single female are expected to share a single mitochondrial haplotype [27]. However, in practical giant grouper breeding, the occurrence of transsexuality is expected to confuse the derivation of mitochondrial haplotypes. Such transsexual fish represents a problem, since these events may cause misidentification of the parent. However, the inclusion of the six selected microsatellite loci is expected to resolve this shortcoming. To determine sibling relationships between any two individuals, microsatellite loci of one parent must be known. However, there is no microsatellite loci database for grouper broodstock.

## 3. Experimental Section

### 3.1. Fish Samples

**Figure 1:**
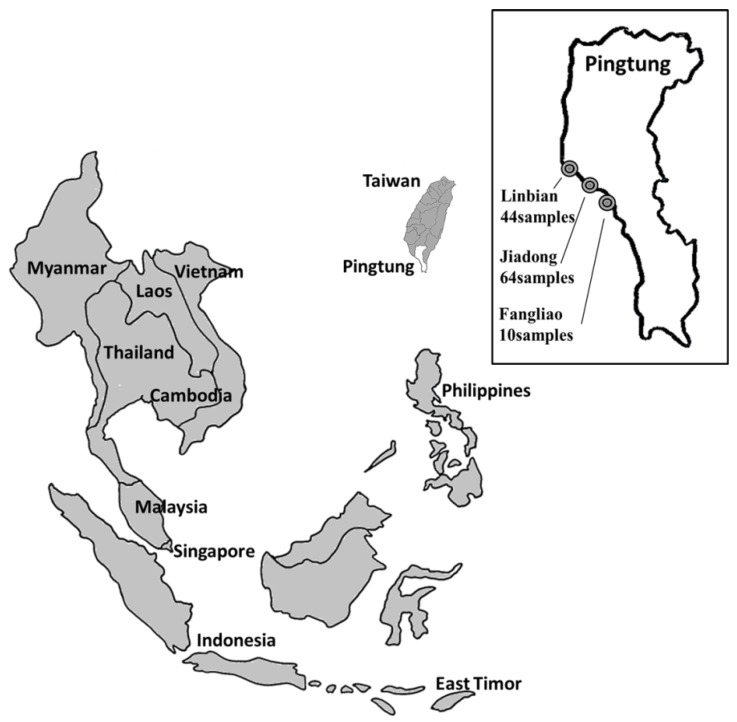
Giant grouper samples collected from farms in Pingtung region, Taiwan. Map (Modified from a map [28] under a Creative Commons Attribution-ShareAlike 1.0 License) inset shows locations of three different farms used for sampling: Linbian (*n* = 44), Jiadong (*n* = 64), and Fangliao (*n* = 10), for a total of 118 fish.

The fin tissue samples from 118 individual broodstock animals were collected in three different locations (Linbian (44 fish), Jiadong (64 fish), and Fangliao (10 fish); Figure 1). Each fish in the same fishing pond was implanted and tagged with radio frequency identification (RFID) chips.

### 3.2. DNA Extraction

A piece of fin tissue (100 mg) sampled from each grouper was subjected to the following DNA extraction procedure. The fin tissue was homogenized in 1 mL of extraction buffer (10 mM Tris-HCl, pH = 8.0, 2 mM EDTA, 10 mM NaCl, 10 M DTT, 1% SDS) with 100 μg mL^−1^ of proteinase K, then incubated at 55 °C in a water bath until the tissue was completely dissolved. After lysis was completed, 20 μL of RNaes A (10 μg/mL) was added to the tube, and then incubated at 37 °C for 1 h. DNA extraction was performed using the phenol-chloroform (phenol:chloroform:isoamyl alcohol = 25:24:1) method. Following precipitation in 99% ethanol, air dry and resuspension in TE buffer (10 mM Tris-HCl, pH = 8.0, 2 mM EDTA).

### 3.3. Establishment of Haplotype Database

Full-length (16,642 bp) giant grouper mitochondrial DNA, as sequenced by our laboratory (accession number: KJ451389), was used for primer design. The forward (ATCATCGGCCAAATCGCATC) and reverse (GAACTGTAGGGCATTCTCAC) primers were designed to amplify a 1259-bp fragment. We then focused on the 776-bp D-loop sequence. Genetic analysis, including nucleotide composition, mutation rate, nucleotide diversity (π) [29], haplotype diversity (Hd) [30] and genetic distance (Kimura’s two-parameter model), were performed using MEGA 5.1 [31] software. DAMBE 5.3.10 [24] software was used to analyze the transition/transversion ratio of D-loop mutations.

### 3.4. PCR and Analysis of Microsatellite Loci

The selection of the potential microsatellite loci as genetic markers in our species was based on previous studies. Primer design focused on several loci described in previous studies for various species (Table 2), including *Epinephelus lanceolatus* [26], *E. guttatus* [23], *E. fuscoguttatus* [25], *E. coioides* [20], *E. quernus* [21] and *E. awoara* [32]. The PCR reaction consisted of an initial round at 94 °C for 5 min, followed by 30 cycles of 94 °C for 1 min, 55 °C for 1 min, and 72 °C for 1 min, with a final extension at 72 °C for 10 min. In order to get a better resolution, ten microliters of the PCR product then was analyzed by electrophoresis on a 12% polyacrylamide gel at 100 volts for 75 min and visualized by ethidium bromide. Following clean-up, the PCR products were sequenced using an ABI PRISM 3730 DNA analyzer (Life Technologies Co., Carlsbad, CA, USA).

The number of alleles for each locus, distribution of alleles, observed heterozygosity (OH), expected heterozygosity (EH), and agreement with Hardy-Weinberg equilibrium was measured using MSA 4.05 [33] software. Cervus 3.0.3 [34] software was used to calculate the combination of non-exclusion probability of the loci as parentage identification. The primers used in this study are listed in Table 2.

## 4. Conclusions

So far, there are about 300 male giant grouper broodstocks in Taiwan. Collection the genetic information from microsatellite to develop markers for each broodstock will be crucial for addressing the problem of inbreeding. For the techniques described in the present work, the mitochondrial D-loop should be used first to determine the potential sibling relationships among fish; biogeographic analysis then can be used to identify the individual broodstock to reduce inbreeding. Furthermore, the marker can be used to preclude the offspring from parent fish which shared the same microsatellite pattern but different in mitochondrial DNA sequence. In fact, the numbers of male broodstock typically are much lower than those of female broodstock, so maintenance of a separate male broodstock (along with genetic screening) may constitute an effective way to manage the genetic variation of farmed giant grouper.
